# Impact of body mass index on breast cancer in accordance with the life-stage of women

**DOI:** 10.3389/fonc.2012.00123

**Published:** 2012-10-04

**Authors:** Reiko Suzuki, Shigehira Saji, Masakazu Toi

**Affiliations:** ^1^Division of Medical Nutrition, Department of Healthcare, Tokyo Health Care UniversityTokyo, Japan; ^2^Department of Target Therapy Oncology, Kyoto University Graduate School of MedicineKyoto, Japan; ^3^Department of Breast Surgery, Kyoto University Graduate School of MedicineKyoto, Japan

**Keywords:** BMI, body weight, breast cancer, estrogen receptor, progesterone receptor, risk

## Abstract

A large amount of epidemiological evidence suggests that the impact of body weight on breast cancer risk should be heterogeneous throughout the life-stage of women. At birth, high weight has been positively associated with an increased risk of breast cancer. While, the body mass index (a relative body weight; BMI kg/m^2^) has been inversely associated with breast cancer risk among pre-menopausal women. The inverse trend had been observed in both Western and Asian population, with a relatively lower percentage of obesity and higher percentage of leanness, suggested that the inverse trend could be explained not only by the protective impact due to obesity, but also by the increased risk of breast cancer due to leanness. Among post-menopausal women, however, an elevated BMI has been positively associated with the development of breast cancer, particularly in the cases of estrogen receptor-positive (ER+) and progesterone receptor-positive (PR+) tumors. Currently, the mechanisms underlying the heterogeneous impacts between BMI on breast cancer risk and the life-stage of women remain poorly understood. We reviewed several proposed biological mechanisms that may contribute to the various impacts of relative body weight on breast cancer risk across life-stage. We also discussed the impact of BMI upon the outcome of endocrine therapy, particularly for aromatase inhibitor, in breast cancer patients. To prevent breast cancer incidence and recurrence, the desirable BMI of women may differ throughout their life-stage. To define the underlying mechanism for the various impacts of BMI in the context of breast cancer across various female life stages, further studies will be required.

The heterogeneous impact of body weight on breast cancer risk has been reported across the female life stages (Bergstrom et al., 2001; IARC Working Group on the Evaluation of Cancer-Preventive Strategies, 2002). High birth weight (>4,000 g/8.5 lb) has been positively associated with an increased risk of breast cancer according to the latest results of meta-analysis (Xu et al., 2009). Although not in all cases (Renehan et al., 2008), protective inverse associations between high body mass index (a relative body weight; BMI kg/m^2^) and breast cancer risk have been suggested among pre-menopausal women (Weiderpass et al., 2004; Li et al., 2006; Michels et al., 2006a). Following menopause, it has been found that a substantial positive association exists between BMI and the development of breast cancer risk, particularly in hormone receptor-positive tumors (Suzuki et al., 2009).

The underlying mechanisms of these heterogeneous impacts between BMI on breast cancer risk and female life stage remain to be fully elucidated. Several plausible biological mechanisms for these various impacts across life stages have been proposed, including hormone-related mechanisms, as an increased BMI could affect levels of steroid hormones such as estrogens and progesterone. These hormones are known to act as important mitogens for mammalian epithelial cells (Anderson and Clarke, 2004) and can mediate their morphogenic effects upon the mammary gland through paracrine signaling (Brisken et al., 1998; Mueller et al., 2002; Mallepell et al., 2006). Growth factor-related mechanisms have also been proposed.

Here, we review the impact of relative body weight upon breast cancer risk across women’s life stages. We study females at birth as well as at pre-menopausal and post-menopausal stages. Additionally, we discuss the impact of BMI on the outcome of endocrine treatment of breast cancer patients.

## BIRTH WEIGHT

Many of previous epidemiological studies had reported a positive association between birth weight and breast cancer risk. The results of meta-analyses (Michels and Xue, 2006; Xu et al., 2009) and a recent population-based, case–control study among Asian-Americans (Wu et al., 2011) provided a substantially increased risk of breast cancer with excess birth weight. The magnitude of the summary of risk estimate according to a dose–response meta-analysis is a 7% increased risk (95% CI: 2–12%) per each 1-kg increase in birth weight (Xu et al., 2009). Although the underlying explanation has not been elucidated yet, one of the most plausible biological mechanisms is estrogen-related one. Heavier birth weight may be a proxy indicator of intrauterine exposure to high levels of estrogen hormones (Trichopoulos, 1990; Mucci et al., 2003), known to be associated with risk of breast cancer development (Pike et al., 1993; Anderson and Clarke, 2004). Previous epidemiological studies, including the Nurses’ Health Study (Michels et al., 2006b; Hurley et al., 2011), have reported stronger positive associations between birth weight and the development of breast cancer in estrogen receptor-positive (ER+) and/or progesterone receptor-positive (PR+) tumors compared to estrogen-independent tumors. Not all (Vatten et al., 2002), but birth weight has been positively associated with higher intrauterine estrogen (Petridou et al., 1990), and earlier age at menarche (Wu et al., 2011) as well as inversely associated with level of α-fetoprotein (AFP), anti-estrogenic property (Nagata et al., 2006). These results could support the estrogen-related mechanism. Elevation of steroid hormones with increased birth weight (Petridou et al., 1990; Nagata et al., 2006) might stimulate a function of mammary stem cells (MaSC) (Asselin-Labat et al., 2010), and this might be linked to a subsequent risk for the development of breast cancer.

In fetal growth, mitogenic activity of the insulin receptor-mediated insulin action may lead to promotion of tumor growth. It has also been reported that a positive link exists between growth factors and birth weight (Vatten et al., 2002). Other proposed mechanisms include (i) a joint effect of insulin-like growth factor 1(IGF-1) and estrogens on breast cancer risk (Schernhammer, 2002; Vatten et al., 2002); (ii) a potential link between loss of genetic imprinting of IGF-2, which could play a role in fetal growth (Constancia et al., 2002) and breast cancer risk (McCann et al., 1996; Wu et al., 1997; van Roozendaal et al., 1998). Further, a function of MaSC should be considered (Asselin-Labat et al., 2010). It was suggested that MaSC are highly sensitive (Shackleton et al., 2006) to steroid hormone signaling, in spite of a lack of ER/PR (Asselin-Labat et al., 2006); (iii) an increase of the number of susceptible MaSC or the stimulation of tumor initiation through DNA mutations due to excess levels of growth factors (Michels and Xue, 2006).

## PRE-MENOPAUSAL WOMEN

In the early-adult stage (i.e., pre-menopausal), epidemiological results have revealed a weak, or in some cases, a substantial inverse association between BMI and breast cancer risk (Ursin et al., 1995; Lahmann et al., 2004; Weiderpass et al., 2004; Michels et al., 2006a); these results include cohort studies among Chinese (Li et al., 2006) and Japanese women (Kawai et al., 2010; Suzuki et al., 2011). In the Western population, the observed inverse (i.e., protective) association could be partly explained by obese-related preventive mechanisms, such as more frequent anovulatory cycles (Key and Pike, 1988) and a faster clearance rate of estrogens in the liver (Siiteri et al., 1982) due to pre-menopausal obesity. These obese-related preventive mechanisms, however, cannot explain the epidemiological results among the Asian population, which has a lower prevalence of obesity compared to Western populations (Murakami et al., 2009). For example, in the Japanese population, a substantial inverse association between level of BMI at age 20 and breast cancer risk has previously been reported in two prospective cohort studies (Kawai et al., 2010; Suzuki et al., 2011). This suggests the presence of other favorable biological mechanisms (not obesity-related, but body mass-related) in the early adult life stage.

After puberty, secondary sexual development generally causes the ovaries to grow. A subsequent increase in the secretion of ovarian hormones, including estrogens and progesterone, leads to the normal development of the mammary gland. Estrogens generally act in the development of the breast duct. Progesterone, in addition to estrogen, may also play an important role in the development of mammary gland lobes. Previous animal studies (Sakakura et al., 1976, 1979) suggested that fat tissue within the breast mass may play a critical role in the normal development of mammary gland lobes during the early adult stage, and this process may occur synergistically with ovarian hormones (Shyamala, 1999). For young adult women, insubstantial levels of fat tissue and low levels of ovarian hormones within the breast bud may provide an obstacle to the maturity of mammary glands during the early adult stage.

In the early adult stage, a low BMI may indicate a lack of a mammary fat pad or a deficiency of progesterone, which may promote an accumulation of excessive body fat (Kalkhoff, 1982). For normal differentiation of the mammary gland, sufficient levels of mammary fat pads, progesterone, or both, may be required during early adulthood (Sakakura et al., 1979; Russo et al., 1982, 2001).

The inverse trend between BMI 20 years and breast cancer risk is observed both among Western population as well as Japanese population. In the light of a relatively low proportion of overweight among the Japanese female, the observed inverse trend should not explain that overweight-/obese-related mechanism. The development of breast tumor due to leanness needs to be considered as one of possible explanations.

Previous studies suggested that mammary fat pad might play a number of vital roles in normal growth, differentiation in mammary gland morphogenesis epithelial–mesenchymal interaction according to animal studies. It has been suggested that body fat might play an important role in differentiation a normal mammary development. Recently, Prat and Perou (2009) have proposed that a distinct link exists between the human mammary epithelial differentiation hierarchy and tumor subtype. They suggested that within the human breast, the process of mammary epithelial differentiation hierarchy might start with an undifferentiated ER (-) MaSC that differentiates into progenitors. Through the process of differentiation, the progenitors lead to the formation of mature cells that are derived from differentiated luminal epithelial cells. A previous study reported a significant association between leanness at age 20 and an increased risk of breast cancer with ER (-) PR (-) tumors (Suzuki et al., 2011). This result could be explained by immature differentiation due to a lack of body fat. A low BMI resulted in a lack of breast fat and/or a low level of ovarian hormones in the early adult life stage, leading us to hypothesize that a low BMI is associated with an increased risk of breast cancer. Additional research is needed for the role of body fat in young adulthood.

## POST-MENOPAUSAL WOMEN

After menopause, epidemiological evidence found a substantial positive association between BMI and breast cancer risk (Lahmann et al., 2004). One of the most plausible and classical biological explanations is a female hormone-related mechanism because adipose tissue may be a major source of estrogens (Siiteri, 1987), which are critical mitogens for mammary epithelial cells (Pike et al., 1993; Anderson and Clarke, 2004). Previous studies have provided several potential hormone-related explanations for an increased risk of breast cancer with excess post-menopausal body weight, as follows: (1) increased levels of estrogen production due to aromatization of androgens in peripheral fat tissue (Siiteri, 1987; Key et al., 2003) and (2) decreased production of sex hormone binding globulin due to obesity (Enriori et al., 1986). These hormone-related mechanisms could increase the supply of free bioavailable estrogen to breast tissues. In fact, the observed increased risk of breast cancer with increasing post-menopausal BMI was apparently attenuated after adjustment for bioavailable serum estrogen concentration (Key et al., 2003). The effects of estrogens are mediated through the ER and the PR. A meta-analysis showed that the substantial positive association between post-menopausal BMI and breast cancer risk was confined to ER+ and PR+ tumors (Suzuki et al., 2009). Furthermore, it is well known that the observed positive association of post-menopausal BMI with an increased risk of breast cancer was attenuated among ever-users of post-menopausal exogenous hormones (Suzuki et al., 2009). These results strongly support the estrogen-related hypothesis that the substantially increased risk of breast cancer with an increase in post-menopausal BMI is mainly the result of the associated increase in bioavailable estrogens.

An insulin-related mechanism could also be one possible explanation for the positive association between post-menopausal body weight and breast cancer risk. Obesity leads to hyperinsulinemia with insulin resistance. Excess glucose levels could stimulate the cellular proliferation of breast tumors (Yamamoto et al., 1999).

## IMPACT OF BMI ON THE OUTCOME OF ENDOCRINE THERAPY FOR BREAST CANCER PATIENTS

High BMI and obesity are recognized as independent risk factors for the development of breast cancer, as well as for an increased rate of recurrence after primary disease (Morimoto et al., 2002; Loi et al., 2005; Reeves et al., 2007; Protani et al., 2010). Previous studies have demonstrated a correlation between high BMI and poor survival in patients with primary breast cancer (Petrelli et al., 2002; Calle et al., 2003; Dignam et al., 2003; Whiteman et al., 2005; Majed et al., 2008; Protani et al., 2010; Ewertz et al., 2011).

The impact of BMI on treatment outcome has also been reported in patients receiving endocrine therapy. There have been consistent results indicating that the efficacy of aromatase inhibitors (AIs) vary with BMI; however, the efficacy of tamoxifen is not BMI-dependent. In the ATAC (Arimidex, Tamoxifen, Alone or in Combination) trial, which compared the efficacy of anastrozole against tamoxifen as an adjuvant treatment for hormone receptor-positive post-menopausal breast cancer patients, anastrozole, a non-steroidal AI, was significantly less effective in post-menopausal breast cancer patients with a high BMI, whereas an equal efficacy of tamoxifen was shown across all BMI levels (Sestak et al., 2010). Retrospective analyses of the Austrian adjuvant endocrine trials ABCSG6/6a showed similar results. There was no difference in DFS and OS between overweight (BMI ≥ 25) and normal-weight post-menopausal patients when treated with tamoxifen with or without aminoglutethimide in the first 5 years of ABCSG 6 trial period (Pfeiler et al., 2010). In the ABCSG 6a trial, 856 patients who were disease free at the end of ABCSG 6 trial were randomly assigned to receive either 3 years of anastrozole or no further treatment. In normal-weight patients treated with anastrozole had a significant better DFS [hazard ratio (HR) 0.48, *p* = 0.018] compared to control patients. However, overweight patients derived no benefit from anastrozole regarding DFS (HR 0.93, *p* = 0.68) compared to control patients. Identical results were also observed in pre-menopausal women in the ABCSG 12 trial in which a significant impact of BMI on the efficacy of anastrozole plus goserelin, but not of tamoxifen plus goserelin, was found (Pfeiler et al., 2011). For another non-steroidal AI, letrozole, a retrospective study by Schmid et al. (2000) analyzing advanced breast cancer patient showed that patients with a high BMI (≥ 30) treated with letrozole benefited less than patients with a BMI under 30 kg/m^2^. In a recent report from the German BRENDA-cohort study with 4,636 patients, the group of hormone receptor-positive post-menopausal patients with a normal or intermediate weight showed a non-significant statistical trend toward a survival benefit with an AI (the exact AIs were not specified) compared to tamoxifen [recurrence free survival (RFS): *p* = 0.486; HR = 1.29 (95% CI: 0.63–2.62)], while obese patients (BMI ≥ 30) tended to benefit more from tamoxifen [RFS: *p* = 0.289; HR = 0.65 (95% CI: 0.29–1.45); Wolters et al., 2012]. In contrast, one retrospective study by Michaud et al. (2002) did not find an effect of BMI on survival after therapy with anastrozole.

It is reported that estrogen levels are influenced by body weight, with overweight patients having higher levels compared to normal-weight patients (Hankinson et al., 1995; McTiernan et al., 2003; Mahabir et al., 2006). The conversion of androstenedione to estrone or estradiol by aromatase in adipose tissue is a major source of estrogen in post-menopausal women. Aromatization capacity should be higher in obese patients, and it can therefore be hypothesized that aromatases in the excess fatty tissue cannot be fully inhibited by standard dosages of AI.

However, as opposed to anastrozole and letrozole, in the TEAM trial, in which 5 years of exemestane use was evaluated against 2–3 years of tamoxifen followed by 3–2 years of exemestane, exemestane was found to be more effective in obese patients than tamoxifen, although the difference was not statistically significant and was only observed during the first 2.75 years, when exemestane and tamoxifen were compared directly (Seynaeve et al., 2010). Similar observations were observed in the neo-adjuvant exemestane clinical trial, JFMC 34-0601, in Japanese post-menopausal women. Objective response rates with 6 months of exemestane treatment were 21.7% in low-BMI patients (BMI < 22), 56.0% in intermediate-BMI patients (22 ≤ BMI < 25) and 60.6% in high-BMI patients (BMI ≥ 25), respectively (*p* = 0.01; Takada et al., 2011). In a multivariate analysis, low BMI was an independent negative predictor of clinical response. It is understandable that AIs might be less effective in patients with a lower BMI because of the lower baseline estrogen or aromatization levels in these patients.

From these observations, one can argue that the differences in the efficacy of AIs depending on BMI might depend on the type of AI, i.e., non-steroidal vs. steroidal (**Table 1**). Although the specific mechanism of these differences has not been explained yet, it is clear that the efficacy of the AI varies according to patient BMI; the efficacy of tamoxifen, however, does not depend on BMI. Further study would be warranted to define the mechanism of the effect of BMI on AI efficacy, which could lead to selective utilization of AIs and tamoxifen in post-menopausal women.

**Table 1:** Body mass index (BMI) and outcome in clinical trial with aromatase inhibitor.

Study	Population	Drug	Findings	Data	Reference
ABCSG 12	Pre-menopausal	ANA + GOS	Less effective in higher BMI	DFS; HR vs. normal weight = 1.60 (1.06–2.41) in BMI *> *25	Pfeiler et al. (2011)
ATAC	Post-menopausal	ANA	Less effective in higher BMI	RFS; HR vs. normal weight = 1.30 (0.91–1.85) in BMI 30–35, =1.53 (1.01–2.32) in BMI > 35	Sestak et al. (2010)
TEAM (analysis	Post-menopausal	EXE	More effective in	DFS; HR vs.TAM = 0.99 (0.74–1.31) in BMI	Seynaeve et al. (2010)
in 2.75 years)			higher BMI (N.S.)	18.5-25, = 0.84 (0.62-1.14) in BMI 25-30, = 0.74 (0.52-1.05) in BMI > 30	
JFMC 34-0601	Post-menopausal	EXE	Less effective in	ORR; 21.7% in BMI < 22, 56.0% in BMI 22–25,	Takada et al. (2011)
(neo-adjuvant)			lower BMI	60.6% in BMI > 25	

*ANA, anastrozole; GOS, goserelin; EXE, exemestane, TAM, tamoxifen; HR, hazard ratio; DFS, disease free survival; RFS, recurrence free survival; ORR, overall response rate.*

## CONCLUSION

**FIGURE 1:**
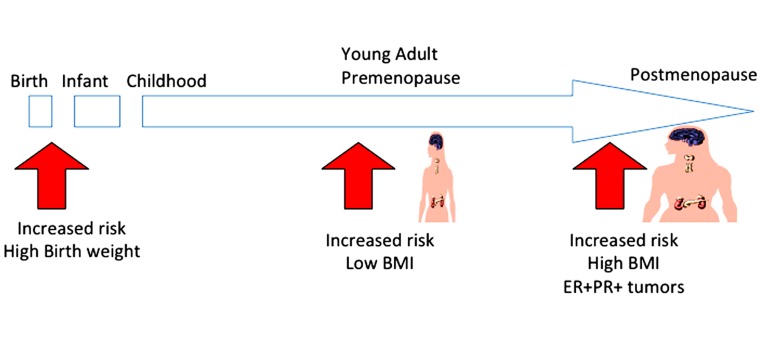
**Impact of BMI on breast cancer risk according to women’s life stage.** Although body weight control is an effective strategy for preventing breast cancer, women’s life stages should be considered. To prevent breast cancer, a low BMI is not recommended during the pre-menopausal stage; a high BMI is not recommended during the post-menopausal stage.

In summary, the current epidemiological study has provided evidence of the impact of body weight on breast cancer risk across women’s life stages (**Figure 1**). Although body weight control is an effective strategy for primary prevention of breast cancer among post-menopausal women, such recommendations should take women’s life stage into account, particularly among young adult pre-menopausal women. In addition, BMI has impact also on the prognosis of breast caner patients and responsiveness to the endocrine therapy with AI. To prevent breast cancer incidence and recurrence, the desirable BMI of women may differ throughout their life-stage. To define the underlying mechanism for the various impacts of BMI in the context of breast cancer across various female life stages, further studies will be required.

## Conflict of Interest Statement

The authors declare that the research was conducted in the absence of any commercial or financial relationships that could be construed as a potential conflict of interest.

## Abbreviations

BMI: body mass index
ER: estrogen receptor
PR: progesterone receptor
